# ROSE in Rosai–Dorfman–Destombes (RDD) disease: a cytological diagnosis

**DOI:** 10.1186/s40001-021-00505-x

**Published:** 2021-04-13

**Authors:** Santosh Tummidi, Hemant Kumar Singh, Prudhvinath A Reddy, Manda Sindhura, Navya Kosaraju, Arundhathi Shankaralingappa, Naresh P Kumar

**Affiliations:** 1 Department of Pathology, AIIMS, Mangalagiri, Guntur, A.P. India 522503; 2 Department of General Surgery, AIIMS, Mangalagiri, Guntur, A.P. India 522503; 3 Department of Radiodiagnosis, AIIMS, Mangalagiri, Guntur, A.P. India 522503

**Keywords:** Rapid on-site evaluation, Cytology, Cellblock, Rosai–Dorfman–Destombes, Emperipolesis, Plasma cell

## Abstract

**Background:**

Rosai–Dorfman–Destombes (RDD) is also known as sinus histiocytosis with massive lymphadenopathy (SHML). It is a benign proliferative disorder of histiocytes, affecting lymph nodes, rarely with extra-nodal involvement. Rapid on-site evaluation (ROSE) with fine-needle aspiration cytology (FNAC) can be utilized as a minimally invasive investigation to avoid unnecessary surgery of this self-limiting disease.

**Case presentation:**

A 65-year-old female presented with complaints of bilateral cervical lymphadenopathy since 1 year. Rapid on-site stain with FNAC from bilateral cervical lymph nodes revealed features of Rosai–Dorfman–Destombes (RDD) disease.

**Conclusion:**

FNAC with rapid on-site evaluation can provide a simple and cost-effective method for looking at the unique cytological features of the disease and act as a first-line investigation.

## Background

Rosai–Dorfman–Destombes (RDD) disease, also called SHML (sinus histiocytosis with massive lymphadenopathy), is an idiopathic lymph node-based histiocytic proliferative disorder. 20–50% of patients with nodal/cutaneous disease undergo spontaneous remission [1, 2]. The clinical findings in such cases can include painless enlargement of the cervical lymph nodes, fever, leukocytosis, anemia, hypergammaglobulinemia, and elevated erythrocyte sedimentation rate (ESR) [3]. Rapid on-site evaluation (ROSE) with fine-needle aspiration cytology (FNAC) is a cost-effective, rapid method that can be used for cytological diagnosis of RDD [4]. Cytology can virtually obviate the need for biopsy in most cases, due to its classic morphological resemblance to histopathological features.

## Case presentation

**Fig. 1:**
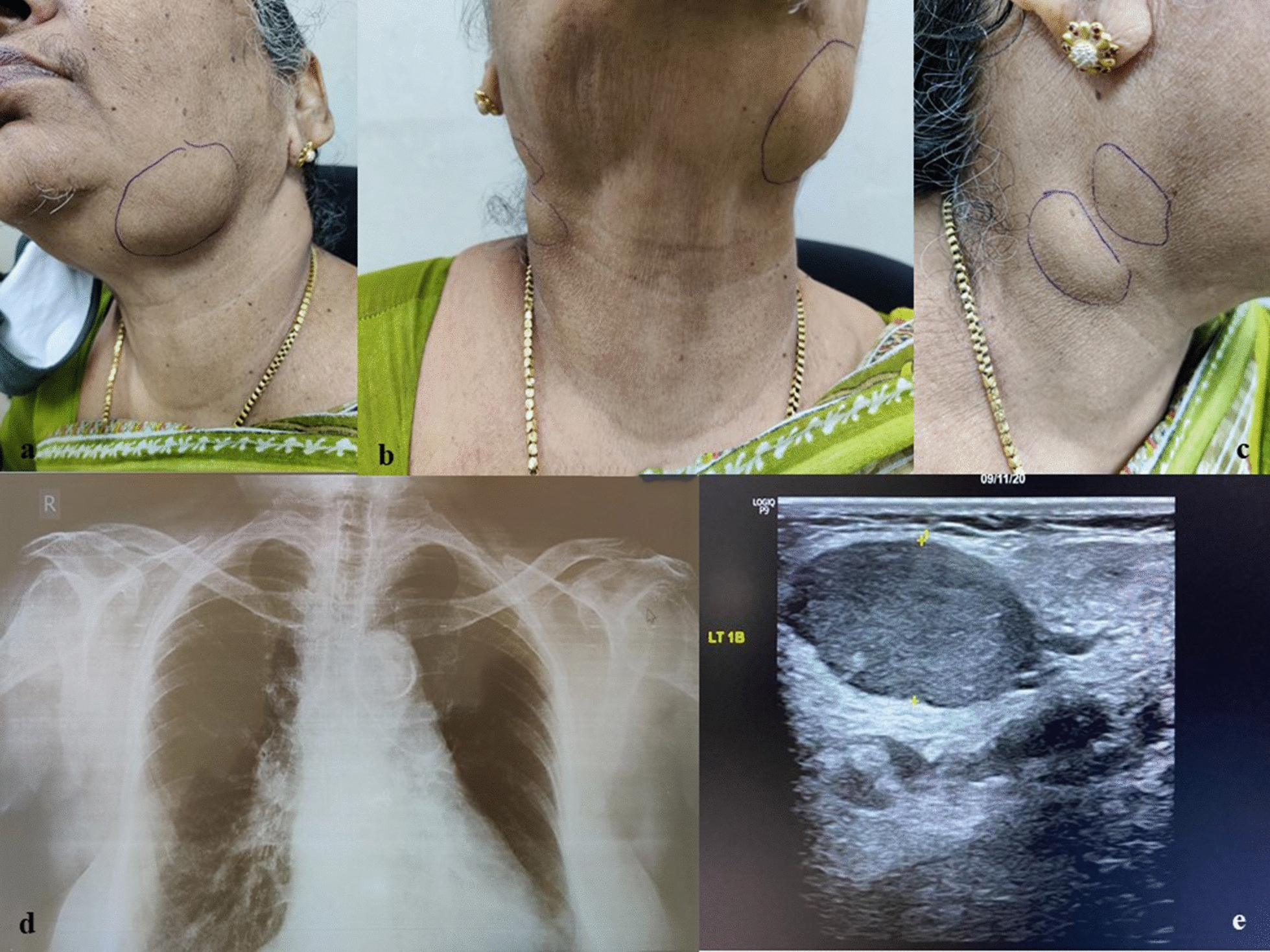
**a**–**c** Patient with multiple matted lymph nodes on left side level Ib, right level IIb, III, and grade I thyroid swelling. **d** Chest X-ray showing bilateral prominent hilar shadow with clear bilateral lung fields. **e** USG neck with level 1b lymph node on left side, hypoechoic and round measuring 2.4 × 1.0 cm

A 65-year-old female presented to our general surgery outpatient department with complaints of bilateral neck swelling of 1 year duration. Her past medical history included cervical carcinoma diagnosed and treated in 1991, hypertension, and coronary artery disease treated with stenting via the external iliac and femoral artery route in 2016. She had no history of night sweats, loss of appetite, weight loss, or evening rise of temperature. She had no other swellings in the body. On examination, she had multiple, bilateral enlarged cervical lymph nodes, including level IB, V on the left side, and level IIB, III on the right. The multiple matted lymph nodes were soft and non-tender on palpitation, with the largest node measuring 2 × 2 cm in the left IB cluster (Fig. 1a–c). She had no significant axillary or inguinal lymph node enlargement. The per-abdominal examination did not reveal hepatosplenomegaly and the oro-pharyngeo-laryngeal examination was unremarkable.

Her serum thyroid profile showed a T3 of 1.02 ng/ml (normal: 0.80–2.0), T4 of 9.9 µg/dl (normal: 5.1 to 14.1), and TSH 3.65 µU/ml (normal: 0.27 to 4.20). Random blood sugar was within normal limits (91 mg/dl). Her albumin was 3.7 g/dl (normal 3.9 to 5.0) and globulin was 5.6 g/dl (normal 2.0 to 3.5). She had an elevated total protein of 9.3 g/dl (normal: 6.5–7.8 g/dl). Her blood hemogram showed a hemoglobin of 8.9 g/dl (normal: 12–15 g/dl), a total leucocyte count of 4660 cells/mm^3^ (normal: 4000–10,000 cells/mm^3^), differential leucocyte count of N56, L34, M07, E03, and B00, and a platelet count of 305,000/mm^3^ (normal: 150,000–410,000/mm^3^). Her peripheral blood picture revealed features of microcytic hypochromic anemia. The erythrocyte sedimentation rate was 100 mm/h (normal: 0–30 mm/h). Her serological markers for hepatitis B, hepatitis C, and human immunodeficiency virus were negative using the lateral-flow card method.

Her chest radiograph showed prominent, bilateral hilar shadows with clear, bilateral lung fields (Fig. 1d). The ultrasound (USG) neck revealed multiple, bilateral enlarged cervical lymph nodes involving levels IB, II, III and IV. The largest lymph node measured 2.4 × 1.0 cm in size and was located in the level 1B group, on the left side. Some of the enlarged lymph nodes were round in shape and hypoechoic (Fig. 1e). USG thyroid showed iso- to hyper-echoic solid nodules measuring 1.1 × 0.6 cm in size, in the left lobe, with peripheral calcification. The right lobe also showed a small sub-centimetric nodule with peripheral calcification. The isthmus was normal. A provisional differential diagnosis of tubercular lymphadenopathy; carcinoma thyroid with possible lymph node metastasis; and lymphoma were considered. FNAC from thyroid and cervical lymph nodes was advised.

**Fig. 2:**
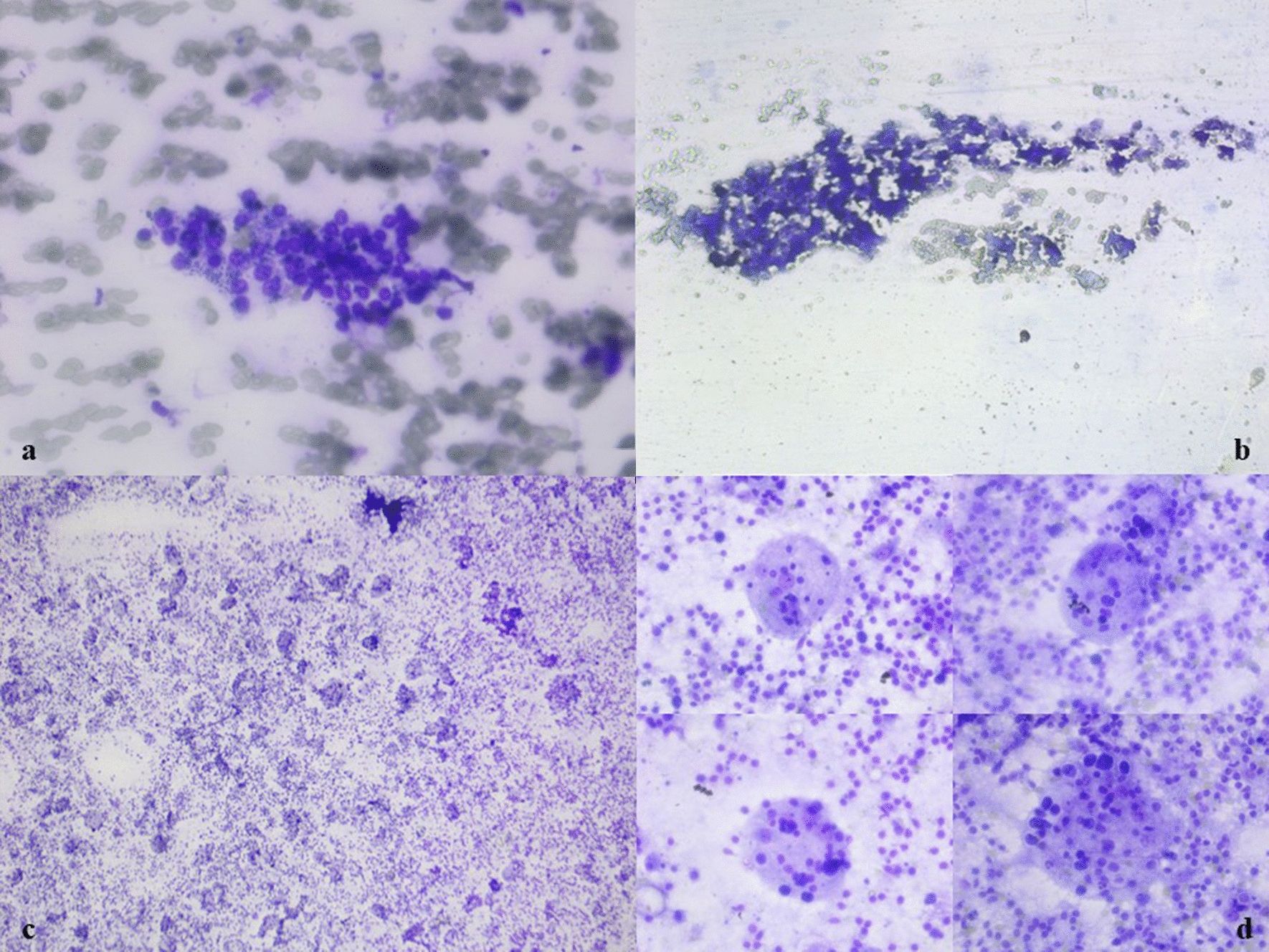
**a** Cytosmears are showing mild cellularity comprising thyroid follicular cells in monolayered sheets with mild nuclear pleomorphism and scant cytoplasm. Few with pigment ladened are also seen. **b** Focal calcification and areas of hemorrhage are also seen [Tol blue, × 40]. **c**, **d** Cytosmears were cellular comprising numerous histiocytes with emperipolesis body having engulfed lymphocytes, plasma cells, RBCs, neutrophils, degenerated cells with abundant eosinophilic cytoplasm and eccentrically placed multiple nuclei. [Tol blue, ×10 and ×40]

Rapid on-site evaluation (ROSE) using 1% aq. toluidine blue solution was employed for the aspirate retrieved from the thyroid swelling (under ultrasound guidance). The specimen showed mild cellularity comprising thyroid follicular cells in monolayered sheets with scattered single cells. The thyroid follicular cells showed mild-to-moderate nuclear pleomorphism with scant cytoplasm. A few Hurthle cells and pigment-laden cyst macrophages were also seen (Fig. 2a). The background showed a thin colloidal material with focal calcification and areas of hemorrhage (Fig. 2b).

**Fig. 3:**
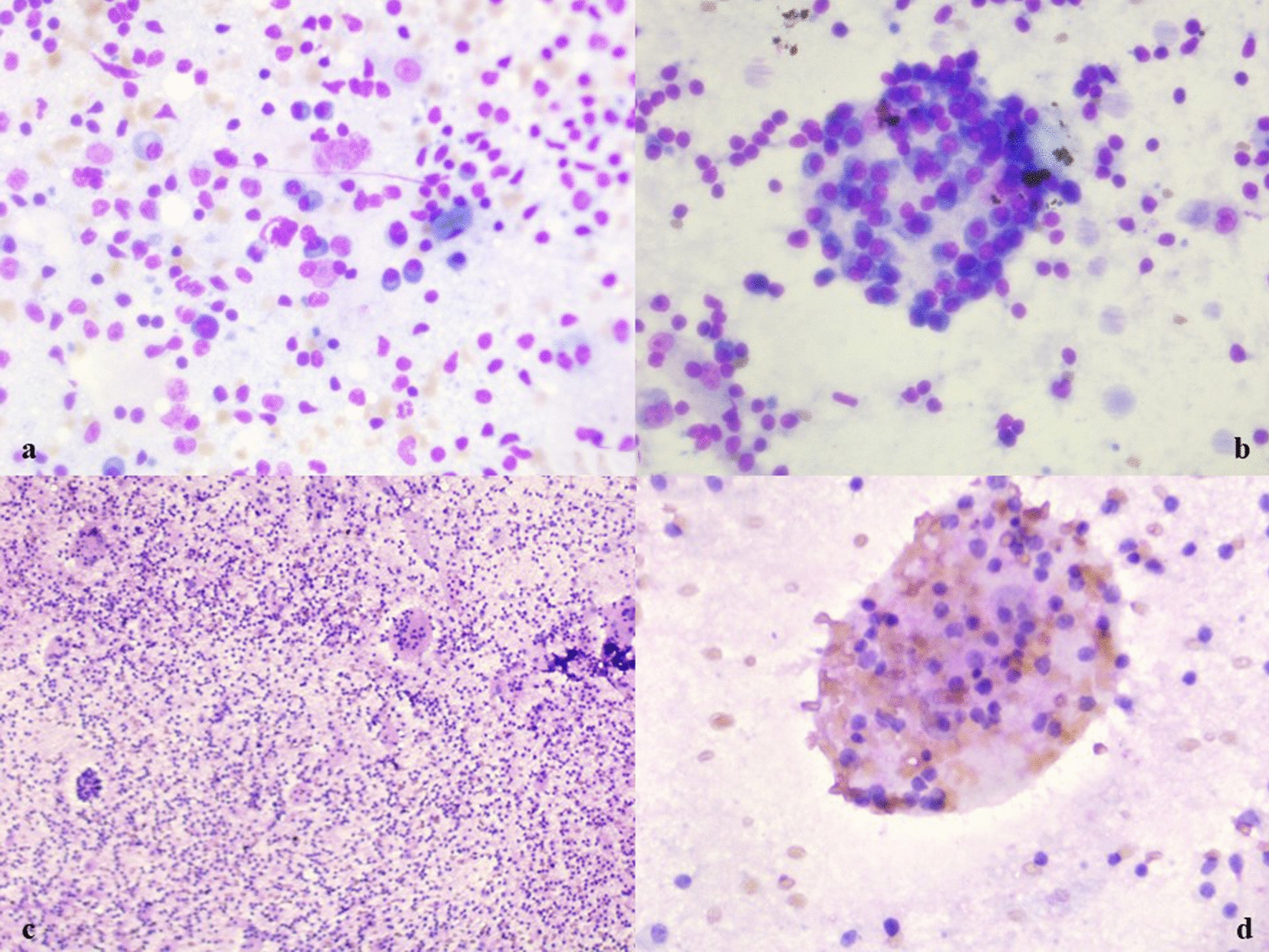
**a**–**d** Cytosmears are showing emperipolesis bodies with lymphocytes, plasma cells, RBCs and neutrophils . The background was showing lympho-glandular bodies with few multinucleated giant cells (Giemsa, ×40 and PAP, ×10, ×40)

ROSE-stained slides from the left cervical level IB and right level IIB groups showed cellular features comprising mature small and large lymphocytes in various stages of maturation. Numerous emperipolesis bodies were also noted with engulfed lymphocytes, plasma cells, red blood cells (RBC), neutrophils and degenerate cells with abundant eosinophilic cytoplasm and multiple, eccentrically placed nuclei (Fig. 2c, d). The slides were then returned for routine cytology staining with Giemsa and Papanicolaou stains. Cytosmears also revealed similar features of emperipolesis bodies with lymphocytes, plasma cells and multinucleated histiocytes. The background showed lympho-glandular bodies with few multinucleated giant cells (Fig. 3a–d). There was no evidence of granuloma or necrosis in the cytosmears. Ziehl–Neelsen stain for acid-fast bacilli was negative. FNAC showed features of Rosai–Dorfman–Destombes (RDD) disease with the thyroid showing colloid goiter with cystic degeneration. Hence, the patient was diagnosed with classical nodal RDD.

**Fig. 4:**
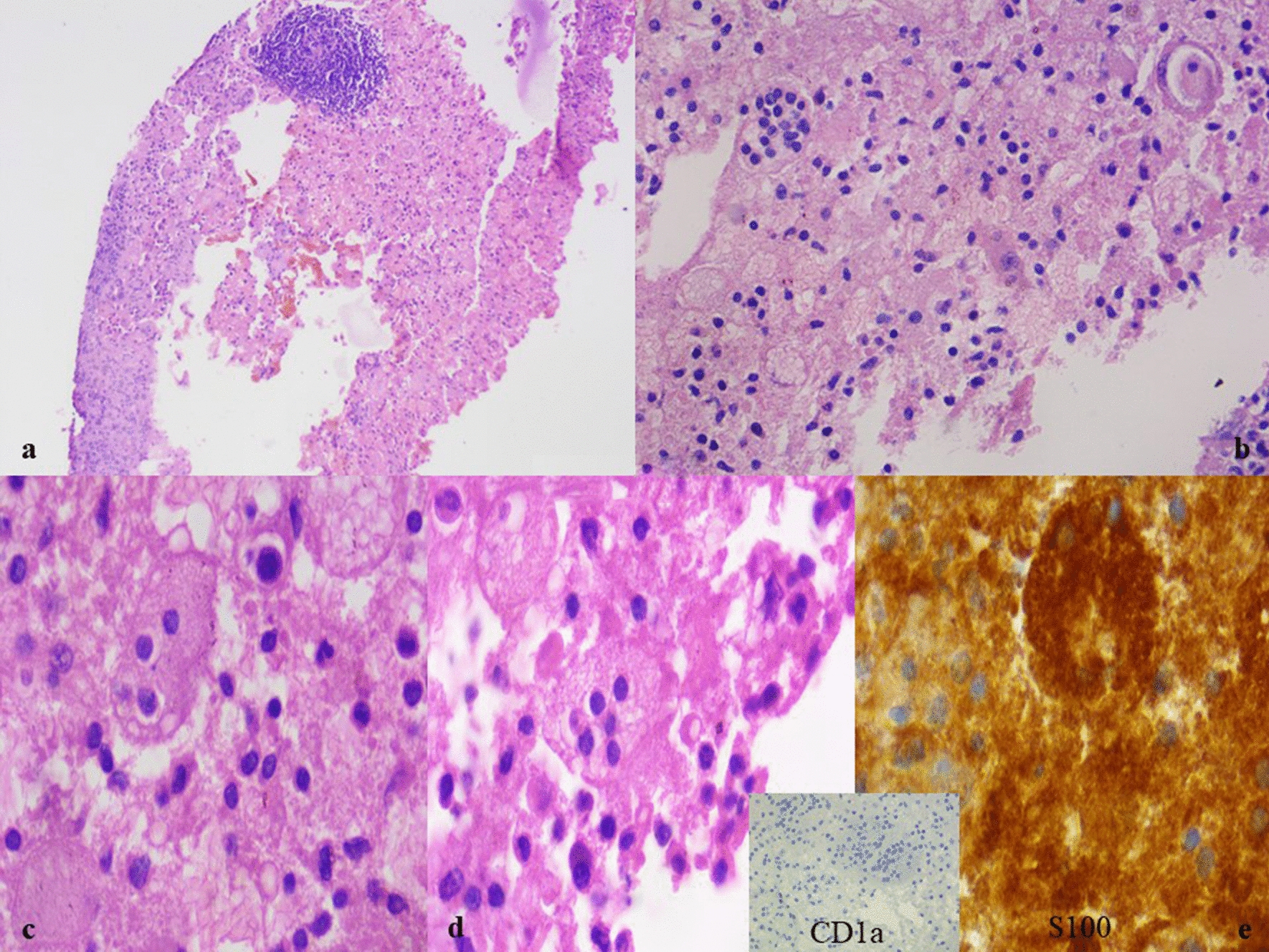
**a–e** Cell block preparation from the lymph node aspirate showing lymphoid follicles with germinal centers along with numerous foamy histiocytes having engulfed plasma cells, lymphocytes and neutrophils. Few scattered plasma cells were also seen in the stroma (H&E, × 10, × 40 and × 100). **e** Immunohistochemistry from the cell block preparation is showing positive for S100 and negative for CD1a (inset) (IHC, × 100)

Cellblock preparation from the lymph node aspirate showed lymphoid follicles with germinal centers replete with foamy histiocytes containing engulfed plasma cells, lymphocytes and neutrophils. Few scattered plasma cells were also seen in the background (Fig. 4a–d). Immunohistochemistry for S100 was positive and CD1a was negative (Fig. 4e).

The patient was started on prednisolone 1 mg/kg/day. After 8 weeks, she showed partial response and the patient is currently on low-dose steroids and follow-up.

## Discussion

RDD is a well-defined clinicopathological entity, first described by Destombes in 1965 [1, 5]. Later in 1969, Juan Rosai and Ronald Dorfman, recognized it as a distinct disease entity comprising sinus histiocytosis and massive lymphadenopathy [5, 6]. 20–50% of RDD patients with nodal/cutaneous disease undergo spontaneous remission [2]. Possible infection and immunodeficiency have been suggested as causes of RDD. Association with HHV6, EBV, *Klebsiella *spp., and CMV has been cited, but no definite etiological link with RDD has been confirmed [6–8]. RDD is also associated with the H syndrome (*SLC29A3* gene), Hodgkin disease, acute leukemia, sarcoma, and immunologic/IgG4 syndrome [2, 9].

RDD is considered to be a self-limiting disorder of an unknown etiology. Clonality studies suggest that lesional RDD cells are polyclonal, reactive, and non-neoplastic. Recent studies identified *NRAS*, *KRAS*, *MAP2K1*, and *ARAF* mutations in patients with features of RDD [2].

Although any age group can be involved, 80% of the cases manifest in the first two decades of life with a male predilection (male:female = 2:1). RDD presents with gradual onset, painless, massive cervical lymphadenopathy, with fever, leukocytosis, elevated ESR, hypogammaglobulinemia, and occasional anemia [1, 10, 11]. Similar findings were noted in our patient. Other lymph nodes, i.e., mediastinal, axillary, inguinal, and para-aortic lymph nodes have also been found affected in RDD. In 40–45% of patients, extra-nodal sites like skin, central nervous system, lungs, cardiothoracic region, subcutaneous tissue, salivary glands, orbits, bone marrow, breasts, thyroid, cervix, and kidneys have also been found affected [12, 13].

Rapid on-site evaluation with fine-needle aspiration cytology can be a useful, cost-effective technique for the diagnosis of RDD [2]. To our knowledge, our case report is the first in the literature to describe ROSE findings of RDD along with the utility of cell blocks for improving the efficacy of FNAC. Aspiration from the lesion showed proliferation of histiocytes with abundant eosinophilic to vacuolated cytoplasm, vesicular single to multiple nuclei, and lymphophagocytosis or emperipolesis in a reactive inflammatory background (lymphocytes in early-stage and plasma cells in later stages). The lymphocytes inside the histocyte have a halo around them, which is not seen in the tissue section due to fixation artifacts [6, 11]. Less often, neutrophils and RBCs can also be seen. FNA may be sufficient to make the diagnosis in most cases thus preventing unnecessary invasive procedures [1, 3, 14, 15].

Histologically, there is an infiltration of the tissue by lymphocytes, histiocytes, and plasma cells. The presence of emperipolesis (histiocytes with engulfed lymphocytes, erythrocytes, and plasma cells) is usually characteristic of Rosai–Dorfman–Destombes disease along with dilated sinusoids [6]. The diagnosis can be confirmed by using immunohistochemical (IHC) markers. Characteristically, S100 is always positive along with other markers, like CD68, CD163, α1 anti-chymotrypsin, and α1 anti-trypsin, with negative results for CD1a and Lagerin (CD207) [11, 12].

The differential diagnoses can include reactive lymph node hyperplasia, infectious lymphadenitis, Langerhans cell histiocytosis, non-Hodgkin's lymphoma, and metastatic carcinoma [3, 12] (Table 1).

**Table 1:** Common differential diagnosis for Rosai Dorfman disease

Disease	Clinical features	Cytology	IHC
Rosai Dorfman disease	Children’s and young adults, M > F, painless lymphadenopathy, extra-nodal presentation seen	Histiocytes with vesicular nucleus and abundant clear cytoplasm, with fine vacuoles and lymphocytes, reactive background of lymphocytes, plasma cells, neutrophils	S100, CD68 positive, CD1a negative
Reactive lymph node hyperplasia	Malaise, painless lymphadenopathies, self-limited disease	Neutrophils, histocytes may or may not be present,	Histiocytes negative for S100
LCH	Localized or multiple lesions with disseminated disease. Nodal involvement may be sole manifestation, bone lesion may be seen	Polymorphic infiltrate with eosinophils and histiocytes with cleaved nucleus	CD1a positive
Hemophagocytic lymphohistiocytosis	May be associated with malignancy of hematological origin, multi organ failure, pancytopenia, Hepatosplenomegaly	Benign histiocytes with engulfed platelets and RBCs	CD68 positive
Non-Hodgkin's lymphoma	Lymphadenopathy, B symptoms weight loss, fever, loss of appetite	Monotonous population of lymphoid cells	Depends of cell of origin
Hodgkin lymphoma	Lymphadenopathy with B symptoms	Polymorphic population with small lymphocytes, eosinophils, plasma cells, and RS cells	RS cell positive for CD15 and CD30
Metastatic carcinomas	Primary in any organ	Lymphoid population with metastatic tumor cells resemblance to primary organ morphology	Depends on organ of origin

RDD has no specific treatment since some patients undergo spontaneous resolution [10]. Surgery may be performed in cases with obstructive/compressive symptoms to vital organs, airway, or with cosmetic issues [5]. Other modalities such as chemotherapy, corticosteroids, low-dose interferon, antibiotics, and radiotherapy have been attempted with variable results. However, the best treatment for RDD is yet to be established [1].

## Conclusion

The cytological features of RDD are so distinctive that they can be diagnosed by FNAC. Implementation of ROSE provides added benefit for collecting samples for cell blocks and IHC, hence obviating the need for invasive investigations. Clinicians and cytopathologists should have a high degree of suspicion for RDD in patients with massive bilateral lymphadenopathy.

## Data Availability

All the data regarding the findings are available within the manuscript

## Abbreviations

CD: Cluster of differentiation
CMV: Cytomegalovirus
EBV: Epstein–Barr virus
FNAC: Fine needle aspiration cytology
HHV: Human herpes virus
RDD: Rosai–Dorfman–Destombes
ROSE: Rapid on-site evaluation
USG: Ultrasonography
